# The role of mindfulness training in sustaining weight reduction: retrospective cohort analysis

**DOI:** 10.1192/bjo.2022.602

**Published:** 2022-11-15

**Authors:** Petra Hanson, Maria Lange, Dominic Oduro-Donkor, Emma Shuttlewood, Martin O. Weickert, Harpal S. Randeva, Vinod Menon, Regi T. Alexander, Paul Basset, Rohit Shankar, Tom M. Barber

**Affiliations:** Division of Biomedical Sciences, Warwick Medical School, University of Warwick, UK; Warwickshire Institute for the Study of Diabetes, Endocrinology and Metabolism, University Hospitals Coventry and Warwickshire NHS Trust, UK; and NIHR CRF Human Metabolism Research Unit, University Hospitals Coventry and Warwickshire NHS Trust, UK; Warwickshire Institute for the Study of Diabetes, Endocrinology and Metabolism, University Hospitals Coventry and Warwickshire NHS Trust, UK; Division of Biomedical Sciences, Warwick Medical School, University of Warwick, UK; Warwickshire Institute for the Study of Diabetes, Endocrinology and Metabolism, University Hospitals Coventry and Warwickshire NHS Trust, UK; NIHR CRF Human Metabolism Research Unit, University Hospitals Coventry and Warwickshire NHS Trust, UK; and Centre for Sport, Exercise and Life Sciences, Faculty of Health and Life Sciences, Coventry University, UK; Division of Biomedical Sciences, Warwick Medical School, University of Warwick, UK; Warwickshire Institute for the Study of Diabetes, Endocrinology and Metabolism, University Hospitals Coventry and Warwickshire NHS Trust, UK; NIHR CRF Human Metabolism Research Unit, University Hospitals Coventry and Warwickshire NHS Trust, UK; and Aston Medical Research Institute, Aston Medical School, Aston University, UK; Adult Learning Disability Services, Hertfordshire Partnership University NHS Foundation Trust, Little Plumstead Hospital, Norwich, UK; and School of Life and Medical Sciences, University of Hertfordshire, UK; Statsconsultancy Ltd, UK; Cornwall Institute of Intellectual Disability Research (CIDER), Peninsula Medical School, University of Plymouth, UK and Cornwall Partnership NHS Foundation Trust, UK

**Keywords:** Complimentary therapies, education and training, psychosocial interventions, cognitive behavioural therapies, outcome studies

## Abstract

**Background:**

Psychological stress has an established bi-directional relationship with obesity. Mindfulness techniques reduce stress and improve eating behaviours, but their long-term impact remains untested. CALMPOD (Compassionate Approach to Living Mindfully for Prevention of Disease) is a psychoeducational mindfulness-based course evidenced to improve eating patterns across a 6-month period, possibly by reducing stress. However, no long-term evaluation of impact exists.

**Aims:**

This study retrospectively evaluates 2-year outcomes of CALMPOD on patient engagement, weight and metabolic markers.

**Method:**

All adults with a body mass index >35 kg/m^2^ attending an UK obesity service during 2016–2020 were offered CALMPOD. Those who refused CALMPOD were offered standard lifestyle advice. Routine clinic data over 2 years, including age, gender, 6-monthly appointment attendance, weight, haemoglobin A1C and total cholesterol, were pooled and analysed to evaluate CALMPOD.

**Results:**

Of 289 patients, 163 participated in the CALMPOD course and 126 did not. No baseline demographic differences existed between the participating and non-participating groups. The CALMPOD group had improved attendance across all 6-monthly appointments compared with the non-CALMPOD group (*P* < 0.05). Mean body weight reduction at 2 years was 5.6 kg (s.d. 11.2, *P* < 0.001) for the CALMPOD group compared with 3.9 kg (s.d. 10.5, *P* < 0.001) for the non-CALMPOD group. No differences in haemoglobin A1C and fasting serum total cholesterol were identified between the groups.

**Conclusions:**

The retrospective evaluation of CALMPOD suggests potential for mindfulness and compassion-based group educational techniques to improve longer-term patient and clinical outcomes. Prospective large-scale studies are needed to evaluate the impact of stress on obesity and the true impact of CALMPOD.

Mindfulness is our human ability to be fully aware of the present moment experience.^1^ In the fast-paced modern world, mindfulness can be difficult to achieve, with many people defaulting to a state of mindlessness.^1^ Mindfulness has gathered momentum in clinical practice, with a growing body of evidence for its application in psychotherapy, pain management and psychiatric disorders.^2,3^ The positive impact of mindfulness on changing eating behaviours in obese populations attending specialist weight management clinics is established.^4,5^

## Obesity and mental health

Obesity is at the forefront of contemporary public health challenges. In 2016, The World Health Organization estimated that 13% of the worldwide population were obese, equating to over 650 million adults.^6^ In the UK, 27% of men and 30% of women live with obesity.^7^ Rates of obesity further increased during the current COVID-19 pandemic, with the monthly rate of body mass index (BMI) increase during the pandemic being 1.93 times that of the pre-pandemic rate.^8^ This is most likely related to less healthy behaviours, such as unhealthy snacking and reduced activity.^9^

Obesity is very closely associated with mental health issues and stress. Evidence showed that people living with obesity had a 30–70% risk of developing mental health issue over their lifetime.^10^ Separate to obesity links to mental health problems, 80% of patients diagnosed with schizophrenia, bipolar disorder or depression were found to be overweight or obese, highlighting a greatly increased prevalence compared with the general population.^11^ A similar bi-directional association between mental health issues and obesity was found in other large studies.^12–14^ The association of stress with unhealthy eating habits, as well as severe obesity, has been clearly documented.^15^

In the UK, people with a BMI >40 kg/m^2^ or >35 kg/m^2^ with a medical condition such as diabetes can be referred to a specialist weight management service (also called tier 3). Despite increasing referral rates, non-attendance is a significant problem for weight management services, with 28.1% patients found to attend <50% of their follow-up appointments and 17.1% not attending their initial appointments.^16^ Mental health factors can play a role in missing appointments, as patients with perceived greater emotional impact of their condition were less likely to attend clinical appointments.^17^

## CALMPOD

CALMPOD (Compassionate Approach to Living Mindfully for Prevention of Disease) is an evidence-based group educational course established on principles of mindfulness, such as mindful eating and self-compassion, that has been shown to be feasible in the short term (6 months) and has a positive impact, both in terms of improving eating behaviour and weight loss.^4^ CALMPOD is not based on any specific mindfulness programme, such as mindfulness-based stress reduction or mindfulness-based cognitive therapy. It aims to introduce the concept of mindfulness in relation to weight management by exploring mindful eating habits and how mindfulness practice might help to build self-compassion and cope with distress.

The aim of this study was to explore the long-term feasibility of CALMPOD by assessing its impact on patient engagement with the service, weight and metabolic markers.

## Method

### Intervention

CALMPOD incorporates mindfulness techniques, and its detailed description is available in a previous publication.^4^ It comprises four group sessions lasting 90 min each, and is delivered every 2 weeks over an 8-week period. There are between six and 12 participants in each group. The sessions are delivered by a specialised weight management psychologist and specialist weight management dietitian. The topics for the course are mindful eating, introduction to compassionate mind therapy, biological drivers for weight regain, environmental challenges, and development of mindful and compassionate planning and management for relapse.

### Study design and participants

This was a retrospective data analysis study based in the clinical context of a specialised weight management service (also called tier 3 weight management service) at University Hospitals Coventry and Warwickshire (UHCW) NHS Trust in the UK. The study was conducted with anonymous clinical data collected between 2016 and 2020. All participants were aged >18 years and had a BMI > 35 kg/m^2^. The STROBE guidance was used to report the study (Supplementary File 1 available at https://doi.org/10.1192/bjo.2022.602). All referrals across the 4-year period were offered CALMPOD at onset. Because of the COVID-19 pandemic, all face-to-face activity ceased in March 2020; however, data collection for those who attended CALMPOD at an earlier date continued until October 2020. Data on clinic attendance was taken from the hospital administration system. Six-monthly recorded data of all patients were extracted from clinical records for the duration of 24 months from the time of each person's prior course attendance offer. Data collected included regularity of attendance, weight, haemoglobin A1c (HbA1c) and total cholesterol. Data of those patients who had not wished to attend CALMPOD or who dropped out after the first two sessions was compared with those who attended at least three of the four sessions.

### Ethics

This was a retrospective, data-driven exercise using routinely collected clinical data by clinicians of the team. The project used anonymised pooled data from the centre. No individual patient data was shared outside the direct clinical team (lead author and last author). This work has been approved by Research and Development Department at UHCW and did not need any formal ethical approvals. Data were collected as part of an ongoing service evaluation and registered as such in the organisation; data were collected retrospectively from existing clinical records. We also used the NHS Health Research Authority tool (http://www.hra-decisiontools.org.uk/research/index.html), which confirmed no formal NHS ethical approval was required (Supplementary File 2). No non-organisation author had access to any patient-identifiable information. All patients provided verbal consent to take part in the group educational course, which was part of weight management service.

### Statistical analysis

The *χ*²-test was used for analysis of appointment attendance. Independent student test or *χ*²-test was used to compare baseline characteristics between two groups. To account for the correlation between repeated observations made on participants’ weights, a linear mixed-effects (repeated measures) regression model was used to quantify and draw inferences on the participant data.

Analysis of the weight data was performed with linear mixed models, to allow for the repeat measurements from the same patients over time. An autoregressive correlation structure was used to allow for the order of the measurements over time.

To calculate the rate of change in each group, time was first considered as a continuous variable. The interaction between time and group was included to compare the rate of change in the two groups. Second, time was considered in categories, to quantify the group difference at each time point. For these analyses, the baseline (time 0) values were excluded from the analysis, with the baseline weight included as a covariate in the model. The interaction between group and time was included to obtain the separate effect at each time point.

## Results

### Baseline characteristics

There were 163 patients who attended at least three of the four CALMPOD course sessions and 126 patients who did not, and whose data was used to compare with the participants. The mean baseline weight was 133.1 kg for the CALMPOD group and 137.1 kg for the comparison group. Nearly 75% of patients in both groups were women, and the mean HbA1c for both groups was at a pre-diabetes level of around 45 mmol/mol. A quarter of patients had diabetes in the CALMPOD and comparison groups (24.4% and 26%, respectively). There was no baseline difference of note in these characteristics between the groups (shown in Table 1).

**Table 1:** Baseline characteristics

	CALMPOD group (*n* = 163)	Control group (*n* = 126)	*t*-Test or *χ*^2^-test comparison between groups (*P*-values)
Age, years	46.9 (s.d. 11.3)	44.5 (s.d. 13.4)	Not significant
Gender, female	74.8%	73.8%	Not significant
Baseline weight, kg	133.1 (s.d. 29)	137.1 (s.d. 27)	Not significant
Baseline total cholesterol, mmol/L	4.7 (s.d. 1)	4.8 (s.d. 1.1)	Not significant
Baseline haemoglobin A1c, mmol/mol	44.5 (s.d. 12.7)	45.3 (s.d. 14.9)	Not significant

CALMPOD, Compassionate Approach to Living Mindfully for Prevention of Disease.

### Patient engagement with the service

**Fig. 1:**
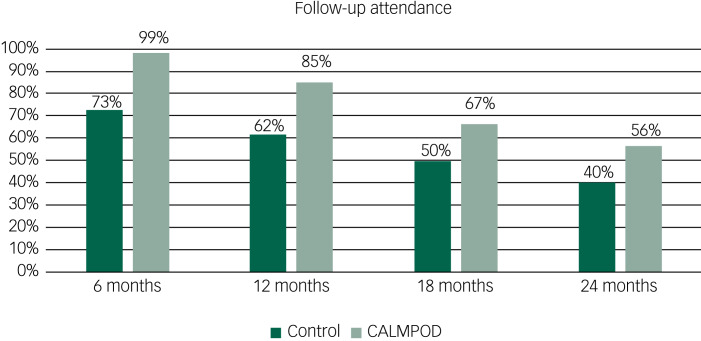
Follow-up attendance.

There was a marked difference between CALMPOD and comparison groups in follow-up attendance. The CALMPOD group was much more likely to engage with the service and attend regular appointments, with 99% (161 out of 173) attending their 6-month follow-up and 56% (92 out of 163) attending their 2-year follow-up; this was significantly more than the 73% (92 out of 126) and 40% (51 out of 126) attendance in the control group. *χ*²-Test analysis showed that the difference between groups was significant at all four time points, with *P* values of <0.001, <0.001, 0.004 and 0.007 for months 6, 12, 18 and 24, respectively. Visual representation can be found in Figure 1. However, when the trajectories of attendance rate over 24 months between the two groups were compared including terms for time and group, along with the interaction between these two terms in the mixed model, no statistical difference between groups in terms of changes over time was identified (*P* = 0.63).

### Changes between groups

**Fig. 2:**
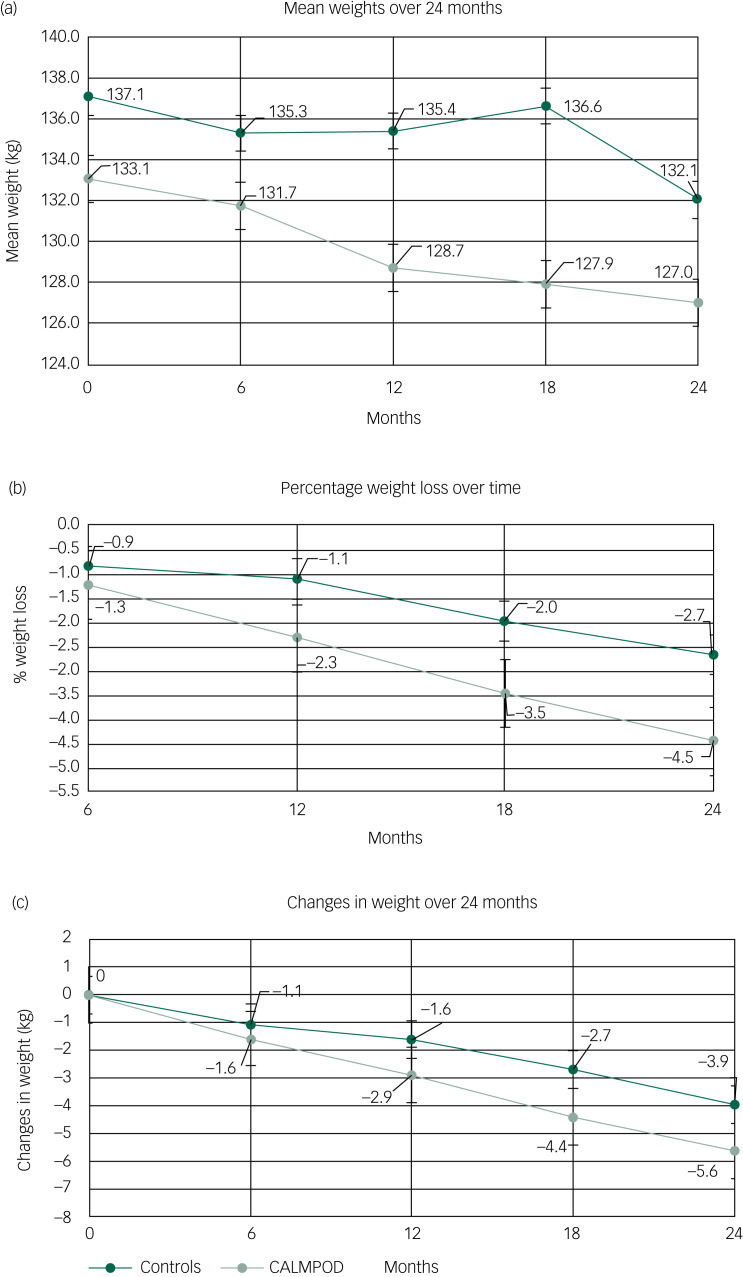
Weight changes over time, with bars representing s.e. of the mean.

There was no statistically significant difference between the groups in terms of starting weight, age, baseline HbA1c and lipid levels. Mean weight loss was 5.6 kg (s.d. 11.2) and 3.9 kg (s.d. 10.5) at 24 months for the CALMPOD and comparison groups, respectively, with an absolute difference of 1.7 kg between the two groups. There was a statistically significant weight loss over time in both group participants. However, the rate of weight loss was not statistically different between the CALMPOD and comparison groups (*P* = 0.939). The mixed-model analysis showed that the gradient of weight loss over time was 0.22 kg per month (*P* <0.001) in the CALMPOD group and 0.17 kg per month (*P* ≤ 0.001) in the comparison group. Based on the mixed-model analysis, the calculated difference in weight loss between the groups was 1.2 kg at 24 months. Weight changes over time are shown in Figure 2.

To calculate the rate of change in weight over time, time was included as a continuous variable in the regression model. The non-linearity of the time effects was investigated by including higher-order terms for time (quadratic and cubic terms). These did not improve the fit of the regression model. This finding, along with visual plots of the data, suggested no evidence that there was a non-linear relationship between weight and time. The second model of using time in categories was also explored, and there were no significant results to report (Table 2).

**Table 2:** Weight changes over time

Time point	Control group			CALMPOD group			Difference^a^	*P*-value
	*n*	Weight	Weight change	*n*	Weight	Weight change	Mean (95% CI)	
Baseline	126	137.1 ± 27.0	−	163	137.1 ± 29.0	−		
6 months	92	135.3 ± 29.6	−1.1 ± 6.5	161	131.7 ± 29.6	−1.6 ± 5.4	−0.7 (−2.7, 1.4)	0.53
12 months	78	135.4 ± 25.8	−1.6 ± 7.7	139	128.7 ± 29.9	−2.9 ± 7.9	−1.2 (−3.3, 0.9)	0.27
18 months	66	136.6 ± 28.8	−2.7 ± 9.6	109	127.9 ± 30.0	−4.4 ± 9.2	−1.4 (−3.7, 0.8)	0.21
24 months	51	132.1 ± 24.2	−3.9 ± 10.5	92	127.0 ± 31.9	−5.6 ± 11.2	−1.2 (−3.7, 1.3)	0.34

Summary statistics are presented as mean ± s.d. CALMPOD, Compassionate Approach to Living Mindfully for Prevention of Disease.

a. Difference reported as weight in the CALMPOD group minus weight in the control group. Differences adjusted for weight at baseline.

The reduction in HbA1c was 0.91 mmol/mol and 0.01 mmol/mol in the CALMPOD and control groups, respectively; however, the difference was not statistically significant. Similarly, there was not a significant difference in total cholesterol change between the groups, with 0.22 mmol/L and 0.18 mmol/L reduction in the CALMPOD and control groups, respectively.

## Discussion

This is, to our knowledge, the first study investigating the long-term impact of mindfulness-led eating and compassion-based techniques incorporated into a group education course among patients attending a specialised obesity service. We found that a mindfulness and compassion-based course is associated with improvements in long-term clinical follow-up rates and leads to a significant weight loss over time. It is worth highlighting that there was significant weight loss over time in the control group as well.

A growing evidence base exists to support the use of mindfulness and compassion-based techniques in clinical practice. A recent meta-analysis of 21 randomised controlled trials of third-wave cognitive–behavioural therapies that use mindfulness concepts, such as mindfulness-based cognitive therapy and compassion-focused therapy, found that the intervention contributed to greater weight loss than standard behavioural treatment.^18^ This analysis found that the weight loss difference was 0.6 kg immediately post-intervention and 1.4 kg at the 24-month follow-up.^18^ This is similar to our study findings of 0.7 kg at 6 months and 1.7 kg at 24 months. The evidence supports incorporation of mindfulness techniques into clinical practice, both for improvements in patient engagement with the service and long-term weight outcomes.

The mean weight in the control group at month 18 was higher than at months 6 and 12, but the percentage weight change and changes in weight at month 18 was much lower than at months 6 and 12 (Fig. 2). The reason for this is the different numbers of participants in the analysis at the different time points. For example, there were 78 participants in the control group with data at 12 months, but only 66 at 18 months. In the control group, the baseline weight of patients with data at 12 months was 137.0 kg, but at 18 months it was 139.3 kg. Thus, because of the different baseline values within these two subgroups, the actual weight at 18 months was higher than at 12 months, but the reduction in weight was greater.

### Limitations of the study

Limitations of this study include its observational nature, small sample size, lack of psychological assessments and its design as a single site study. Given its nature as a retrospective observational study, confounding and bias effects, such as changes to patients’ medications, education, social status self-selection and motivation, would have played role. It could be argued that there was participant bias as those more motivated to lose weight were more engaged with the course. However, this project was an explorative study to identify suitable associations for further inquiry, and not causality. Further, the same clinicians delivered the care in the control group, and were therefore likely to be offering the same advice and support as in the group, on an individual level. However, patients attending the CALMPOD group had more interactions with the clinical team, which could have fostered more motivation and contributed to improved attendance rates. We did not measure changes in mindfulness level and long-term eating behaviour changes, nor was an inquiry made into baseline psychological levels, socioeconomic status or education levels.

Finally, it is not clear to what extent the results were confounded by the effect of the global COVID-19 pandemic. However, as most of the data collection occurred pre-pandemic, it is likely that only a small proportion of the data were directly affected by the pandemic. Nevertheless, it is clear that the pandemic contributed to worsening obesity rates, and the impact of this will continue to be observed in years to come.

### Implications for clinical practice

The mainstay of obesity management is lifestyle changes, including increasing physical activity and reducing caloric input.^19^ Albeit effective in the short term, the evidence of sustained weight loss following these interventions is poor, with over a third of weight being regained within the first year.^20^ The challenge for the individual who is aiming to sustain weight loss is in complying with behaviours that counteract their own physiology.^21^ The onus should be on clinicians to provide their patients with the tools to develop effective and sustainable behavioural modifications that go further than instructions to simply reduce calorific intake. Obesity management should be focused on preparing the patient mentally for the challenges of a healthy lifestyle change. The fact that patients engaged better with the service after the CALMPOD intervention is an important outcome for both patient and health services.

### Implications for research

In this retrospective, data-driven observational study, we assessed 24-month outcomes of the mindfulness-based educational course CALMPOD in a clinical setting of a specialised weight management service. The results of this study suggest a positive association of such a course on patient engagement, as well as significant weight loss over time, with little resource expenditure. To provide robust evidence on the effectiveness of such interventions, multi-site randomised controlled trials need to be set up. Additionally, measures of mindfulness scores and validated questionnaires on eating behaviours and psychological health (e.g. depression, anxiety) should be collected. To further explore factors predictive of non-attendance, variables such as educational level, marital status and socioeconomic status should be collected, as they have been shown to affect rates of non-attendance in previous studies.^22^

## Data Availability

Anonymised data that support the findings of this study are available from the corresponding author, R.S., upon reasonable request.

## Supplementary material

For supplementary material accompanying this paper visit http://doi.org/10.1192/bjo.2022.602. click here to view supplementary material
